# Pathways to Parkinson’s disease: a spotlight on 14-3-3 proteins

**DOI:** 10.1038/s41531-021-00230-6

**Published:** 2021-09-21

**Authors:** E. Giusto, T. A. Yacoubian, E. Greggio, L. Civiero

**Affiliations:** 1 IRCCS San Camillo Hospital, Venice, Italy; 2 Center for Neurodegeneration and Experimental Therapeutics, Department of Neurology, University of Alabama at Birmingham, Birmingham, AL USA; 3 Department of Biology, University of Padova, Padova, Italy

**Keywords:** Cellular neuroscience, Diseases

## Abstract

14-3-3s represent a family of highly conserved 30 kDa acidic proteins. 14-3-3s recognize and bind specific phospho-sequences on client partners and operate as molecular hubs to regulate their activity, localization, folding, degradation, and protein–protein interactions. 14-3-3s are also associated with the pathogenesis of several diseases, among which Parkinson’s disease (PD). 14-3-3s are found within Lewy bodies (LBs) in PD patients, and their neuroprotective effects have been demonstrated in several animal models of PD. Notably, 14-3-3s interact with some of the major proteins known to be involved in the pathogenesis of PD. Here we first provide a detailed overview of the molecular composition and structural features of 14-3-3s, laying significant emphasis on their peculiar target-binding mechanisms. We then briefly describe the implication of 14-3-3s in the central nervous system and focus on their interaction with LRRK2, α-Synuclein, and Parkin, three of the major players in PD onset and progression. We finally discuss how different types of small molecules may interfere with 14-3-3s interactome, thus representing a valid strategy in the future of drug discovery.

## Introduction

14-3-3s define a ubiquitous, highly conserved family of proteins whose name reflects the elution fraction of an ion-exchange chromatography performed by Moore and Perez in 1967, in which 14-3-3s were discovered to be major components of bovine brain lysates. Since then, 14-3-3s have been identified in all eukaryotes studied so far, spanning from unicellular organisms to mammals, although differences in their isoform-specific expression and function have emerged along the evolutionary process^1^. Most of the known organisms express multiple isoforms of 14-3-3s (indicated with Greek letters), each encoded by a distinct gene^2^. Despite early observations suggesting functional overlap, later reports established that different isoforms have acquired a certain degree of specificity (reviewed in ref. ^3^). The biological relevance of 14-3-3s has remained underrated for over 20 years, until they emerged as crucial components in the biosynthesis of neurotransmitters in the brain^4^. Almost at the same time, multiple lines of evidence showed that 14-3-3s could orchestrate different biological activities within the cell, including cell cycle progression^5^, cell transformation and mitogenic signal regulation^6,7^, intracellular signaling^8,9^, and exocytosis^10^. However, it was not until 1996 that crystallographic studies began to reveal the hallmark structure of 14-3-3s and, with this, some powerful insights into their polyhedric nature^11^. In particular, it was discovered that 14-3-3s were present mainly as homo- or heterodimers and that they were capable of binding numerous target proteins containing specific phospho-serine/phospho-threonine motifs^11^, although many exceptions also exist^12^. The ability of 14-3-3s to target such an exceptional range of “client” proteins represents the essence of their multitasking functionality and explains their critical involvement into multiple cellular activities.

Parkinson’s disease (PD) offers a unique platform in this context. Indeed, 14-3-3s stand at the crossroad of several pathways involved in the pathogenesis of the disease. For example, 14-3-3β and 14-3-3γ were found to be at the center of some transcriptionally deregulated pathways in PD patients^13^, while isoforms -γ and -η show the strongest affinities for leucine-rich repeats kinase 2 (LRRK2), a key protein in the onset of familial and sporadic PD (see below)^14^. Likewise, 14-3-3η has been shown to regulate the ubiquitin ligase activity of Parkin and is able to reduce the formation of toxic α-synuclein aggregates in vitro, while isoforms -θ, -ε, and -γ exhibit a major neuroprotective effect in experimental models of PD^15–17^. Therefore, a better understanding of 14-3-3s functional network and a selective modulation of their interactions with crucial partners may represent a new strategy to disentangle the molecular roots of PD.

### Origin and evolution

Multiple numbers and combinations of 14-3-3 isoforms are present in distinct classes of organisms, ranging from budding (*S.pombe*) and fission (*S.cerevisiae*) yeasts, which both express two isoforms^18^, to *Arabidopsis*, which expresses up to 15 different isoforms^6^. The mammalian genome host seven major transcripts (β, γ, ε, ζ, η, θ/τ, σ) encoded by distinct genes, while those originally described as α- and δ- isoforms have finally been recognized as the phosphorylated forms of β- and ζ- 14-3-3s, respectively^19,20^. Studies based on sequence alignments suggest the presence of a common ancestor from which different isoforms would have diverged along with the evolution of major eukaryotic lineages. Within the animal group, the -ε isoform holds the most ancestral sequence, and shares some key features with yeasts and plants, thus diverging from the remaining non-ε isoforms^21^. Interestingly, some isoforms may share higher sequence identity (up to 96–100%) with paralogs belonging to different species within the same group (e.g., mammalian) rather than with other isoforms in the same species (about 50%)^1^.

The biological relevance of such a variety is controversial. Early studies describing an efficient replacement between 14-3-3 isoforms coming from different species seemed to support the hypothesis of functional redundancy, as expected also by the high degree of sequence homology. Indeed, the expression of plant or human-derived 14-3-3s effectively rescued the lethal phenotype caused by the double disruption of the yeast genes *BMHI* and *BMH2* (coding for 14-3-3s in *Saccharomyces cerevisiae*)^22^. Yet, the existence of isoform-specific motifs as well as a preferential tissue or cellular tropism and a differential subcellular recruitment, suggest that a substantial degree of functional specialization does exist^3^. More recently, the combination of high-throughput analyses with the availability of databases hosting considerable amounts of protein–protein interaction (PPI) data, has pointed to the concept of “adaptive subfunctionalization”. According to this, mild mutations may damp the deleterious effects of dosage-imbalance caused by duplication events, thus reconciling genetic redundancy and functional specialization^23,24^.

### Structural characteristics of 14-3-3s

**Fig. 1:**
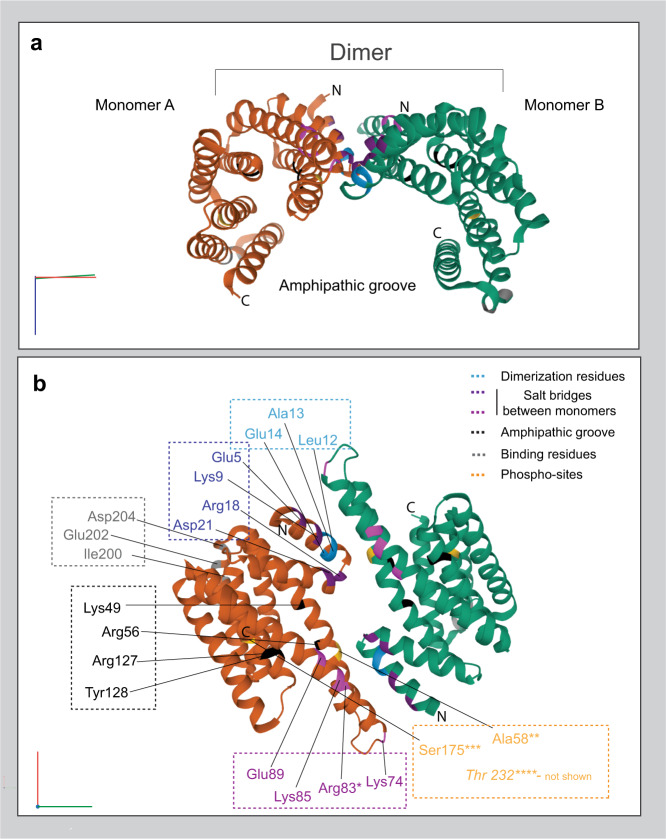
Structure of 14-3-3. Orthogonal views of human 14-3-3θ/τ, modified from 6BD1^226^. **a** Schematic representation of the main structure of human 14-3-3θ/τ showing the spatial allocation of the amphipathic groove. **b** Some of the most significant residues involved in structure stability and functionality. Some isoform-specific mutations are depicted: Arg83* is replaced by Glu83 in the 14-3-3σ isoform and involved in Lys9-Glu83 binding. Ala58** is present only in isoforms -θ/τ and -σ; all the other isoforms present a Ser58 which is a very important phosphorylation site (see main text). Ser175*** indicated as Ser184/186 in the main text. C: C-terminal, N: N-terminal.

In homeostatic conditions, 14-3-3s exist mainly as dimers (Fig. 1a), a configuration that is believed to favor their stability (e.g., by reducing susceptibility to phosphorylation) and activity^25^. However, dimers can be dissociated and converted into monomers upon the phosphorylation of specific residues (see below), and certain 14-3-3 isoforms may naturally exist as monomers and dimers^26,27^. Also, a defective splicing variant of 14-3-3ε which lacks the N-terminal region—usually involved in dimer formation—has been recently found^28^.

All 14-3-3 isoforms show significant structural homologies, which in part reflect the overall degree of sequence identity. Each monomer is composed of 9 α-helices arranged in antiparallel order and connected by short loops^29^. In dimers, helices from both monomers interact in a symmetrical manner, in which helix α1 (amino acids 2–16) of one monomer connects with helices α3 (amino acids 38–66) and α4 (amino acids 73–103) of the opposite monomer and vice versa^30^. While some isoforms prefer assembling into heterodimers, others mainly form homodimers. This difference depends on the presence of 3 main salt bridges involving Arg18-Glu89, Glu5-Lys74, and Asp21-Lys85 (Fig. 1b). As an example, the -ε isoform only contains one of these salt bridges. Therefore, its association with a different isoform (which inevitably contains more salt bridges) would provide higher stability to the resulting dimer than the one deriving from the association with another -ε monomer. On the other end, the σ-isoform, where the Glu5-Lys74 salt bridge is replaced by the Lys9-Glu83 interaction, is more liable to form homodimers^31^. This aptitude of different monomers to interact further extends the repertoire of target proteins that can be engaged by 14-3-3 dimers. The conformational organization of dimers results in a cup-shaped pocket approximately 35 Å broad, 35 Å wide, and 20 Å deep, meant to accommodate the target protein(s)^32^. Structure analyses of 14-3-3s bound to peptides have revealed that the side chains of some specific residues, namely Lys49, Arg56, Arg127, and Tyr128, confer an amphipathic nature to the groove, crucial to the binding of target proteins (Fig. 1a, b)^1,32,33^. These residues outline the most inner part of the pocket and are strictly conserved in mammals and yeasts^29^. They form numerous hydrophobic and polar interactions, as well as salt bridges between either identical or different isoforms, thus conferring a rigid structure to the dimer, essential for its function^34^. Of note, these conserved residues give each isoform the same potential to bind a peptide, while variable residues that line the external surface of the groove provide the specificity of each 14-3-3:target interaction^32^. Despite the high degree of sequence homology at the level of the groove (over 70% between organisms)^35^, N- and C-terminals are quite divergent between isoforms, with the main exception being represented by ^12^LAE^14^ residues, which are conserved in all the species and crucial for the dimerization process (Fig. 1b)^26,36^.

Intriguingly, because of some peculiar variations in the amino acidic sequence surrounding the binding groove, its total lack of introns, its main expression in epithelial cells, and its preference at forming homodimers, the σ isoform represents the most peculiar member within the 14-3-3 family^37^.

### Definition of consensus binding motifs

The high level of sequence conservation of the amphipathic groove suggests that target proteins may also need specific anchoring residues to be recognized and accepted at the dimer interface. While some recurrent binding motifs have been determined, the repertoire of target proteins able to interact with 14-3-3s is much more extended than initially thought. Early observations suggested that the phosphorylation of a Ser residue might have been pivotal. In support of this, Michaud et al. showed that the phosphorylation of Ser259 in the CR2 domain of Raf-1 was strictly required for its interaction with 14-3-3s, which could have been prevented by phosphatase treatment or by the mutated S259A-Raf^38^. Similarly, endogenous phosphorylation of tryptophan hydroxylase by a Ca^2+^/calmodulin or cAMP-dependent kinase is essential for its binding to 14-3-3η in vitro^39,40^. By using a panel of degenerated peptides derived by c-Raf, Muslin and collaborators identified RSX(pS/pT)XP as a first putative binding motif recognized by 14-3-3s (mode I). Notably, all the isoforms are able to recognize this motif and bind with high affinity^11^. Similar results were obtained also by Yaffe et al., who, in addition, proposed RX(F/Y)XpSXP as a novel binding motif (mode II)^32^. More recently a third motif (mode III) has been identified. This consensus sequence, pS/TX_1-2_-COOH, lies at the C-terminal of target proteins, and still requires phosphorylation to bind 14-3-3s. Only a few targets containing this motif have been studied so far, and further investigations are needed to better define the most common surrounding residues. Finally, some proteins known to interact with 14-3-3s fall outside these conventional binding rules and do not strictly depend on phosphorylation events^12^.

The presence of two binding sites (one from each monomer) in a 14-3-3 dimer suggests that 14-3-3s may simultaneously bind different targets and function as scaffold proteins. However, it is also true that one single target protein may contain more than one binding site such that one target protein can interact with both 14-3-3 monomers. In many cases, the binding affinity of these additional sites may be too low to ensure a long-lasting interaction, as binding affinity depends on the extent to which the binding motif matches the optimal consensus sequence. Within a target protein, there is likely a dominant binding site that acts as a “gatekeeper” and favors the concomitant interaction of secondary (weaker) binding sites. In fact, an association between 14-3-3s and proteins containing tandem phospho-serine motifs can lead to a 30-fold increase in their binding affinity^32,41^. In this context, Stevers and coworkers propose a thermodynamic model to describe how a delicate enthalpy/entropy balance is crucial in determining the overall binding affinity of a target protein to 14-3-3s. In particular, the authors analyze different, multivalent bindings for cystic fibrosis transmembrane conductance regulator (CFTR) and for the PD multi-domain kinase LRRK2, which present nine and six binding sites for 14-3-3s, respectively. Importantly, they noticed that LRRK2 peptides carrying different combinations of the 14-3-3s binding sites present different *K*_d_ values, with the highest ones shown by pS910 and pS935. Therefore modifications of these sites may be of interest for therapies aimed at blocking or reinforcing 14-3-3:LRRK2 binding (see below)^42^.

### 14-3-3s biological functions

**Fig. 2:**
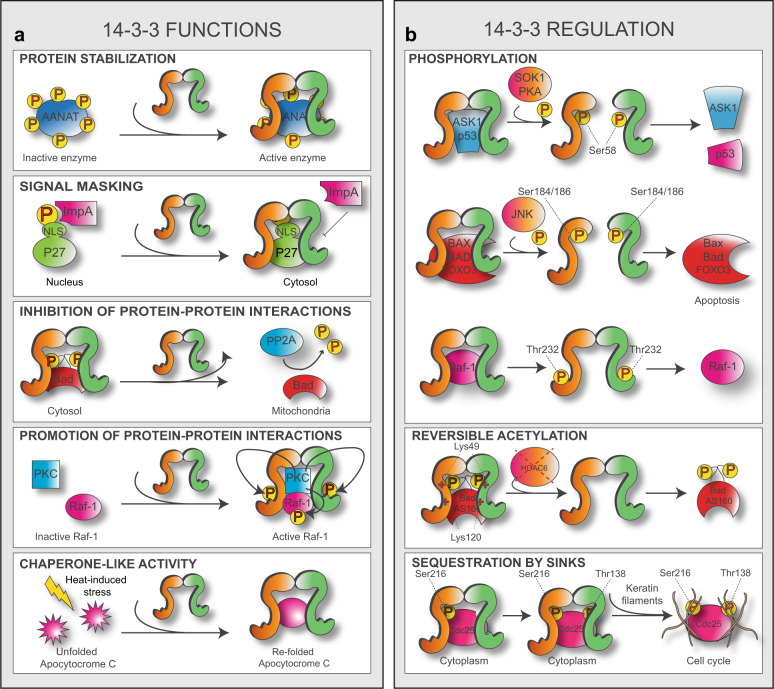
Modifications and modalities of function of 14-3-3. Panels in **a** and **b** show some of the diverse modalities of activity and regulation of 14-3-3s as described in the main text. P: phosphate group; AANAT: serotonin N-acetyltransferase; ImpA: importin α; NLS: nuclear localization signal; BAD: BCL2 associated agonist of cell death; PP2A: protein phosphatase 2; PKC: protein kinase C; Raf-1; ASK1: apoptosis signal-regulating kinase 1; SOK1; PKA: protein kinase A; BAX: BCL2 associated X; FOXO3: forkhead box class O 3; JNK: c-Jun N-terminal kinases; AS160: Akt substrate of 160 kDa; Cdc25.

Because of their rigid structure, 14-3-3s are thought to work as “molecular anvils”, showing little or no change in their overall conformation upon target binding. This interaction would instead force target proteins to undergo changes in their structure, thus altering some of their functional properties, localization, or PPIs^41^. One of the first studies supporting this theory showed that 14-3-3s stabilize the structure of phosphorylated Serotonin Acetyltransferase (AANAT), increasing its catalytic activity and substrate affinity (Fig. 2a, top box)^43^. 14-3-3s can also influence the functionality of target proteins by masking or exposing their nuclear localization motifs. This is particularly important for those proteins whose activity is controlled by their subcellular localization, such as those involved in cell cycle regulation. p27 is a cyclin-dependent kinase inhibitor which can be phosphorylated at multiple sites, including Thr157, a residue contained in its nuclear localization signal (NLS). Once bound to phosphorylated Thr157, isoforms 14-3-3β, -ε and -γ mask this NLS and prevent the interaction between p27 and importin-α, with the consequent cytoplasmic withholding of p27 (Fig. 2a, second box)^44,45^. Alternatively, upon binding to 14-3-3s, target proteins may fail to interact with their downstream partners, as in the case of the regulator of G protein signaling 3 (RGS3)^46^. Similarly, BAD needs to dissociate from 14-3-3s to interact with phosphatase PP2A and initiate the apoptotic program (Fig. 2a, middle box)^47^ and ref. ^2^.

14-3-3s can also act as scaffolds. Thanks to their ability to simultaneously bind different target proteins through their bivalent docking site, 14-3-3s can work as hubs to promote functional interaction between partners. As an example, 14-3-3 isoforms -β and -ζ can form heterotrimeric complexes with Raf-1 and protein kinase C (PKC)-ζ. Once the complex is formed, PKC-ζ can phosphorylate both Raf-1, which becomes active, and 14-3-3s, finally leading to the dissociation of the ternary complex (Fig. 2a, fourth box). Brief, temporary interactions like this may provide a mechanism by which cells activate dynamic signaling cascades in response to external stimuli^48,49^.

Another recognized, but less studied function of 14-3-3s is the one related to their chaperone-like properties. While 14-3-3s do not meet all of the conventional criteria commonly attributed to “professional chaperones”, they do participate in the regulation of protein folding and aggregation^50–53^. Although considered an auxiliary, “moonlighting” activity of 14-3-3s^54^, it gains particular relevance for those pathologies characterized by the formation and accumulation of misfolded proteins, such as Alzheimer’s disease and PD. It has been shown, indeed, that 14-3-3θ and 14-3-3η can prevent the misfolding and aggregation of α-Synuclein both in vitro and in vivo, thus reducing its propagation and toxicity^17,52,55^. This may also explain why 14-3-3s are highly detected in LBs of PD patients^56^. This chaperone-like activity does not rely on the recognition and binding of specific phospho-sequences, and it does not require the existence of either the hydrophobic groove or of the flexible C-terminal stretch^57^. Instead, a prominent role is exerted by the salt bridge interactions lying at the dimer interface, whose dynamic nature strongly impacts on the transient status of disorder at the N-terminal region and on the monomer–dimer equilibrium of 14-3-3s. In particular, mutations of Asp21 or Glu89 are sufficient to alter the N-terminal α-helix order and shift the monomer–dimer balance in favor of the monomeric conformation. Similar to other chaperones, 14-3-3s must recognize and bind hydrophobic regions exposed by unfolded proteins. Therefore, by exposing some key residues, such as those framing the hydrophobic groove and the crucial Ser58 (see below), monomeric forms of 14-3-3s exhibit higher chaperone-like activities compared to their dimeric counterparts^26,34,58^. Nevertheless, evidence of phosphorylation-dependent chaperone-like activity also exists. As an example, Liang and colleagues demonstrated that forskolin-induced phosphorylation of the R region of CFTR favors the binding of 14-3-3β leading to an increase in CFTR expression and stability^59^.

Similar to heat shock proteins, 14-3-3ζ transcription is increased upon heat-induced stress in *Drosophila*, likely due to the presence of a heat-induced transactivator. This results in the dissolution of aggregated apocytochrome c and Hsp70/Hsp40-mediated refolding of citrate synthase, once more suggesting relevant similarities between 14-3-3s and molecular chaperones (Fig. 2a, bottom box)^60^. The identification of the chaperone-like activity of 14-3-3s poses some new intriguing questions in the field. How does this role co-exist with the more canonical phospho-specific-targeting molecular hubs? What are the molecular cues that determine which activity 14-3-3s need to take on? How is chaperone-specificity achieved? Further experiments are needed to shed some light on these crucial questions. However, the observation that one single protein has been given custody of such a variegated repertoire of activities, might suggest a conservation process where modifications of a pre-existing abundant protein might have occurred cheaper and faster, in evolutionary terms, than the de novo creation of many different proteins.

### Regulation of 14-3-3s

The 14-3-3 interactome can be further enlarged by a variety of post-translational modifications (PTMs) (for relevant reviews see ref. ^61^ and ref. ^62^). One of the most common PTMs is phosphorylation. Accordingly, 14-3-3s contain a few critical Ser/Thr residues whose phosphorylation can affect their dimerization status and interaction with target proteins. In particular, the invariant residue Ser 58/59 of 14-3-3η, -γ, -β, and -ζ can be recognized by different kinases, leading, in the majority of the cases to 14-3-3s dissociation and reduced functional interaction with their associated partners (Fig. 1b). As an example, p21-activated protein kinase 6 (PAK6), recognizes and phosphorylates Ser59 of 14-3-3γ, thus promoting its dissociation from LRRK2 (see below)^63^. Intriguingly, in both σ and θ/τ isoforms, Ser58 is naturally replaced by an alanine residue, which, by definition, cannot be phosphorylated^64^. Doubts remain on how kinases can reach such a hidden spot, although sphingosine has been shown to bind 14-3-3s and change 14-3-3 conformation, thus rendering Ser58 prone to phosphorylation^30,64^.

Other two residues that can be phosphorylated are Ser/Thr232 and Ser184/186. This latter was initially identified within a consensus motif for proline-directed kinases and believed to belong to 14-3-3α and 14-3-3δ, subsequently recognized to be the Ser184-phosphorylated forms of 14-3-3β and 14-3-3ζ, respectively^19,65^. Ser184/186 of 14-3-3ζ and 14-3-3σ is a target of the c-Jun N-terminal kinase (JNK). Upon phosphorylation, 14-3-3ζ and 14-3-3σ release the pro-apoptotic Bax, which then translocates to the mitochondria and initiates the apoptotic cascade^66^. Phosphorylation of Thr232 inhibits the binding between 14-3-3ζ and Raf-1^67^, likely the result of a conformational change of the C-terminal stretch, which is involved in 14-3-3:target binding and contains Thr232 (Fig. 2b, top box)^68^.

Acetylation of lysine residues is another relevant PTM observed on 14-3-3s^24^. The addition of acetyl groups masks the positive charge of lysine residues, some of which are present in the binding groove and are crucial for the interaction with target proteins. Inhibition of histone deacetylase 6 (HDAC6) activity on K49 and K120 causes the dissociation of 14-3-3ζ from the AKT-phosphorylated GTPase proteins AS160 and BAD, an event that is prevented in mutant forms of 14-3-3ζ in which these lysine residues have been replaced by arginine (Fig. 2b, middle box)^69,70^. The shift between lysine acetylation and deacetylation may represent another dynamic mechanism to regulate the status of the 14-3-3 interactome^70^.

An additional way to regulate 14-3-3s is gene methylation. Many studies have demonstrated a link between hypermethylated CpG-rich regions of 14-3-3σ and cancer^71^. This epigenetic modification leads to a decrease in 14-3-3σ expression and has been observed in a variety of cancers, including breast cancer^72^, hepatocellular carcinoma^73^, and squamous cell carcinoma^74^. The in vitro treatment of cancer cells with 5′-aza-2′-deoxycytidine (an inhibitor of DNA methylation) recovers 14-3-3σ expression^72,75^.

Another way to regulate 14-3-3s functionality is offered by “sinks”, partners which work as molecular magnets to capture 14-3-3s involved in loose interactions and commit them to new target proteins (Fig. 2b, bottom box)^62,76–79^.

The existence of such a wide potential of mechanisms to regulate 14-3-3 interactions with their target proteins provides further emphasis on the dynamic activity of 14-3-3s, which is crucial to their biological role.

### 14-3-3 roles in the brain

14-3-3s were originally described as abundant proteins in the brain (up to 1% of total soluble proteins, and their presence in both neurons and glia has been extensively investigated^80–82^.

**Fig. 3:**
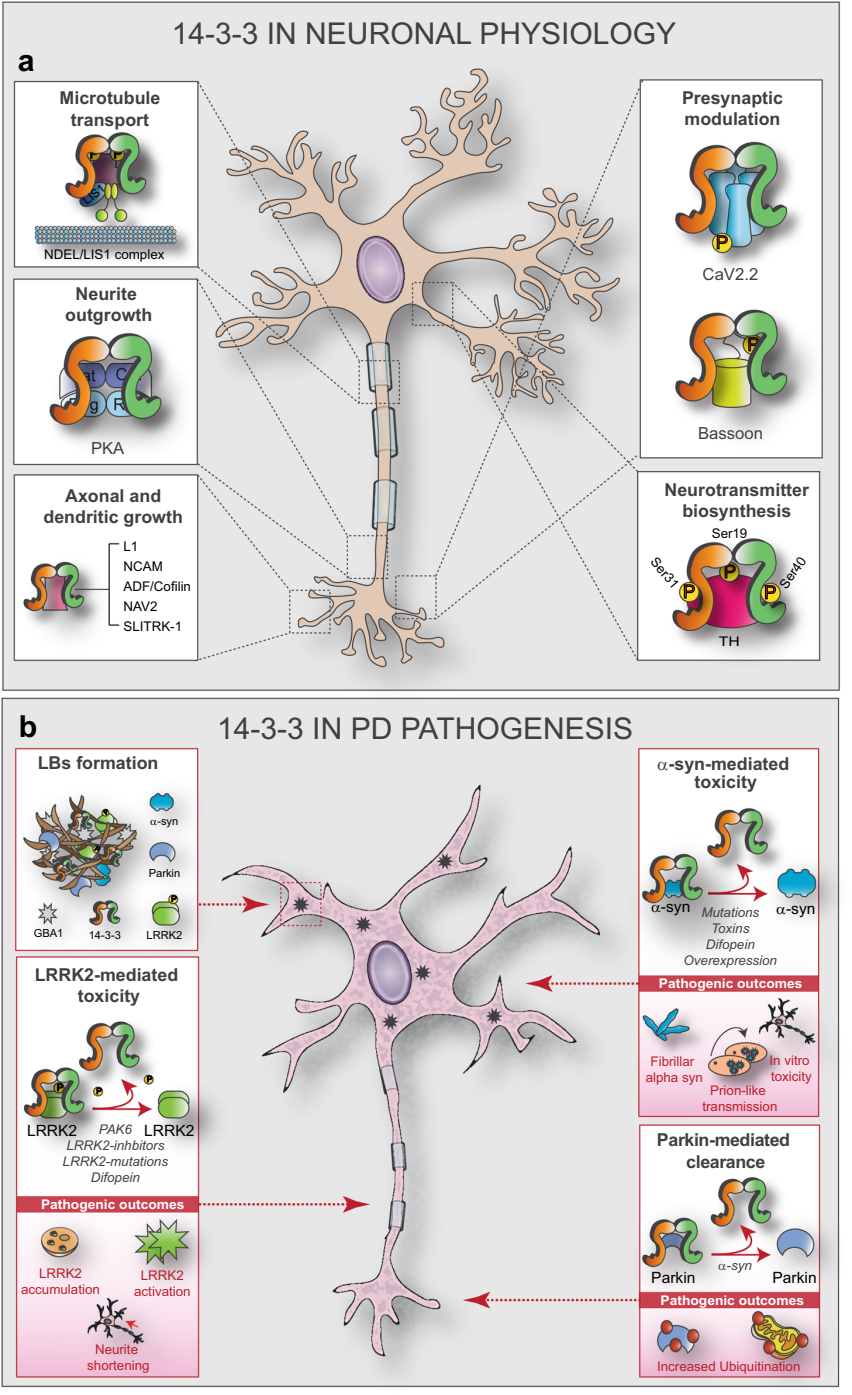
Roles of 14-3-3 in the healthy and PD brain. The roles of 14-3-3s in physiological conditions (**a**) and its involvement in PD (**b**). **a** NDEL/LIS: nuclear distribution gene E-like homolog 1/Lissencephaly-1; PKA: protein kinase A; NCAM: neural cell adhesion molecule; ADF/Cofilin: actin-depolymerizing factor/Cofilin; NAV2: neuron navigator 2; SLITRK-1: SLIT and NTRK-like family, member 1; CaV2.2: N-type voltage-gated Ca2^+^ channel; Bassoon; TH: tyrosine hydroxylase. **b** GBA: glucosylceramidase beta; LRRK2: leucine-rich repeat kinase 2; α-Syn: α-Synuclein; PAK6: P21-activated kinase 6.

14-3-3s are involved in several aspects of brain development, such as neurogenesis, neuronal differentiation and migration, neurite outgrowth, and axonal guidance^83–85^. 14-3-3s take part also in the shaping and maintenance of dendritic spines^86,87^, synaptic plasticity^88^, and neurotransmitter release^89^. In particular, -ε and -ζ isoforms are critical in the early stages of brain development. 14-3-3ε binds to and protects the phosphorylated form of nuclear distribution gene E-like homolog 1 (NDEL1). In turn, NDEL1 binds to Lissencephaly-1 (LIS1) and dynein to promote dynein motor function and neuronal migration (Fig. 3a, top left box). The absence of 14-3-3ε exposes NDEL1 to the action of protein phosphatase 2A (PP2A), thus impairing the migration process^90^. 14-3-3ε KO and 14-3-3ε/ζ double KO mice show severe deficits in cortical layer formation and suffer a significant rate of embryonic lethality. Likewise, deletions in the gene coding for 14-3-3ε in humans are responsible for the onset of Miller-Dieker Syndrome, a severe form of Lissencephaly^91,92^. Similarly, lack of 14-3-3ζ affects the structure of the Disrupted-in-Schizophrenia 1 (DISC1)/NDEL1/LIS1 complex, resulting in migration defects and abnormal synaptic connectivity of hippocampal mossy fibers. In agreement, 14-3-3ζ knockdown mice develop cognitive and behavioral impairments resembling those observed in schizophrenic patients^93^. Moreover, the 14-3-3 functional KO mouse, which expresses the pan 14-3-3 competitive peptide inhibitor difopein (dimeric fourteen-three-three peptide inhibitor), shows similar cognitive and behavioral deficits associated with impaired hippocampal long-term potentiation^87^.

14-3-3s influence growth cone morphology by acting on molecular pathways that transfer extracellular cues to cytoskeletal proteins^94^. Several 14-3-3 isoforms are expressed in the developing dorsal root ganglia in chickens and rats, where they affect the direction of the growing axon. As an example, 14-3-3s stabilize PKA catalytic subunits, preventing the phosphorylation of their downstream targets. This results in a different response (repulsion vs attraction) to nerve growth factor gradients (Fig. 3a, middle left box)^95^. 14-3-3s are also involved in axon regeneration as shown with in vitro experiments of axonal damage, and these activities can be controlled with the use of Fusicoccin-A (FC-A). FC-A stabilizes the binding between 14-3-3s and general control non-derepressible 1 (GCN1) to regulate its degradation and promote neurite outgrowth. FC-A also prevents the die-back phenomenon observed in mice upon dorsal hemisection of the spinal cord^96^. 14-3-3s also interact with the phosphorylated Tricornered-dependent kinesin Pavarotti in *Drosophila*. This complex inhibits the microtubule sliding which is necessary for neurite outgrowth, once more suggesting the involvement of 14-3-3s in dendritic elongation^97^. Other studies using the functional 14-3-3 KO mouse have confirmed a role for 14-3-3s in regulating dendritic length and complexity^87,98^. This process involves several mechanisms, including modulation of the cell adhesion molecules L1 and neural cell adhesion molecule, actin-depolymerizing factor, neuron navigator 2, SLITRK-like family member 1, and the Raf-MEK-ERK pathway (Fig. 3a, bottom left box)^94,99–103^.

14-3-3s are highly expressed at synaptic terminals, where they bind phosphorylated target proteins of the presynaptic zone, such as Bassoon or Ca(V)2.2 channel subunits and contribute to synaptic plasticity and transmission (Fig. 3a, top right box)^86,104^. 14-3-3s interact with the glutamate transporter EAAT2/GLT-1, which is in charge of glutamate reuptake at synapses^105^. Notably, GLT-1 expression and glutamate homeostasis are impaired in several models of PD^106^. 14-3-3s also affect the expression of several glutamate receptor subunits, thus suggesting a role in post-synaptic transmission^107–109^.

Moreover, 14-3-3s bind and activate tyrosine hydroxylase (TH), the step-limiting enzyme required for the biosynthesis of catecholamines, including dopamine^4^. TH can be regulated at several levels, including protein synthesis, mRNA stability, and activity. TH harbors three different Ser residues (Ser19, Ser31, Ser40) whose phosphorylation is essential to its functionality. In particular, 14-3-3s bind TH at phosphorylated Ser19. This interaction allows 14-3-3s to stabilize TH and assist in the regulation of both Ser31 and Ser40 phosphorylation (Fig. 3a, bottom right box)^110^.

All together, these observations indicate a significant role of 14-3-3s in the development and function of the central nervous system. It is therefore not surprising that 14-3-3s may also be linked to the pathogenesis of several neurological diseases.

### 14-3-3 roles in Parkinson’s disease

The progressive degeneration of dopaminergic neurons in the *substantia nigra pars compacta*, results in a reduction in dopamine, which is a key neuropathological feature of PD^111–113^. Depletion of striatal dopamine underlies the classic motor symptoms observed in PD, such as bradykinesia, rest tremor, rigidity, and postural instability^113^. Several experimental models have been generated to recapitulate these neuropathological conditions. Among these, the most used are animals treated with toxins that disrupt the nigrostriatal pathway (e.g., 6‐hydroxydopamine), inhibitors of the mitochondrial complex I (e.g., 1‐methyl‐4‐phenyl‐1,2,3,6‐tetrahydropyridine and rotenone), and proteasomal inhibitors (e.g., epoximycin)^114^. Interestingly, about 10% of total PD cases are familial, and several genes are known to be mutated in dominant or recessive forms of PD. Among these, mutations in *SNCA* and *LRRK2* (encoding α-Synuclein (α-Syn) and LRRK2), cause autosomal dominant forms of PD closely resembling sporadic PD, while recessive mutations in *PRKN*, *PTEN-induced kinase 1*, and *PARK7* genes (encoding for parkin, PINK-1, and DJ-1) cause early-onset parkinsonism^115^. Additional genome-wide association studies have pointed to single-nucleotide polymorphism within *LRRK2*, *SNCA*, and *GBA1*, encoding the lysosomal glucocerebrosidase, as risk factors for the development of PD^116^. The discovery of mutations in specific genes contributing to the pathology of PD has led to the development of transgenic models, which recapitulate certain but not all the pathophysiological features present in PD patients^117^.

Brains of PD patients demonstrate Lewy bodies (LBs) and Lewy neurites, abnormal insoluble aggregates containing fibrillar proteins found in the substantia nigra, locus coeruleus, and other brainstem regions along with regions of the cerebral cortex^118,119^. α-Syn, together with ubiquitin, is one of the major protein components of LBs^120^. Recent in-depth ultrastructural analyses have shown that LBs are composed of membranous material originating from vesicles and fragmented organelles including mitochondria, lipids, and lysosomal structures^121^. Moreover, LBs contain a mixture of proteins (including TH, DJ-1, LRRK2, Parkin, PINK-1, GBA1, SPG11/spatacsin^122^, and mitochondria-related proteins) and molecules implicated in the ubiquitin–proteasome system, autophagy, and aggresome formation (reviewed in ref. ^123^). Importantly, 14-3-3s are present in LBs and are known to interact and co-localize with several proteins involved in PD pathogenesis, including LRRK2, α-Syn, and parkin (Fig. 3b, top left box)^124^ or in other neurodegenerative disorders with parkinsonian syndrome such as hereditary spastic paraplegia caused by mutations in *SPG11*^125^. To this regard, we recently observed that PKA-mediated phosphorylation of Ser955 in SPG11/spatacsin is a signal for the binding of 14-3-3η (10.1101/2020.09.09.289009^126^). Whether 14-3-3 dysregulation is a cause or a consequence of LB formation, is still not clear.

Overexpression of 14-3-3θ/τ, -ε, and -γ is protective against neurotoxin models, such as rotenone and MPTP, while difopein exacerbates cell death^16^. Similar results have been obtained in vivo, where 14-3-3θ/τ protects surviving dopaminergic neurons in a model of MPTP-induced toxicity^127^. Neuroprotective effects are instead lost when 14-3-3θ/τ is phosphorylated at Ser232 in neuroblastoma cells, most likely because of the inability of the phosphorylated isoform to inhibit Bax and its downstream targets^128,129^. Of note, 14-3-3θ/τ phosphorylated at Ser232 is increased on the insoluble fraction of brain lysates from PD patients^129,130^. Thus, 14-3-3s co-localization in LBs, their altered phosphorylation in PD models and patients, and their acknowledged neuroprotective effects in several disease models leads to the speculation that 14-3-3s are likely to play a crucial role in the pathophysiology of PD, and their regulation may represent a possible strategy for future therapies.

#### Interaction between 14-3-3s and LRRK2

LRRK2 is a large multi-domain protein belonging to the ROCO family. It includes a characteristic bimodal region composed of a Ras Of Complex (ROC) domain, responsible for GTP binding and hydrolysis, and a C-terminal Of ROC (COR) domain, which works as a dimerization hub^131^. The ROC-COR tandem is followed by a serine/threonine kinase domain regulated by the GTPase activity, implying a complex intramolecular control^132,133^. Several repeats regions flank this tripartite enzymatic core in the following order: armadillo (ARM), ankyrin (ANK), and leucine-rich repeats (LRR) at the N-terminal, and WD40 repeats at the C-terminal, collectively involved in the regulation and localization of LRRK2^134^. Several missense mutations clustered in the catalytic core (N1437H, R1441C/G/H, Y1699C, G2019S, and I2020T among others) are pathogenic with age-dependent penetrance. Together, these mutations cause autosomal dominant forms of PD that account for 4% of familial and 1% of sporadic cases, although these percentages may increase depending on the population (for a detailed review, please refer to^135^). Among these, the G2019S mutation is the most frequently found in familiar PD and results in clinical and pathological features that closely resemble those described for idiopathic patients, including the presence of LBs and the loss of midbrain dopaminergic neurons^136^. In most cases, these mutations alter the biochemical activities of LRRK2. For example, G2019S and I2020T, both located at the kinase domain, increase the phosphorylation activity of LRRK2^137,138^, most likely through the stabilization of its active status^139^. Alternatively, mutations at the ROC-COR domain have a direct effect on ROC by slowing GTP hydrolysis, which affects cellular phosphorylation of LRRK2 substrates by impairing LRRK2 recruitment to signaling compartments or by improper interactions with ROC binding partners^132,140–143^.

LRRK2 sequence includes several phosphorylation sites, some of which (e.g., Ser1292) are LRRK2 autophosphorylation sites and can be used as bona fide functional readouts to monitor its kinase activity^144^. This is remarkably important, considering that the altered phosphorylation of LRRK2 substrates strongly associates with the pathobiology of PD both in experimental and clinical settings^145,146^. Furthermore, the phosphorylation status of LRRK2 crucially influences its interactions with other proteins, among which are 14-3-3s. Several 14-3-3-binding sites, where LRRK2 is constitutively phosphorylated, are spread across the entire structure of the protein, and include Ser860 on the ANK domain, Ser910, Ser935, Ser955, and Ser973 at the N-terminal, Ser1444 within the ROC domain, and the latest identified Thr2524 at the C-terminus^14,147^. In a very interesting work, Stevers et al. examined the binding affinity and crystal structure of 14-3-3γ complexed with different LRRK2 peptides, each containing regions around the phosphorylation sites Ser860, Ser910, Ser935, Ser955, Ser973, and Ser1444. In agreement with previous results obtained by the same and other labs for c-Raf, PKC, and CFTR^148–151^, simultaneous interactions of 14-3-3γ with multiple docking sites within LRRK2 results into synergistic affinity values. In particular, interactions obtained by combining peptides containing two phosphorylation sites selected among Ser910, Ser935, and Ser1444 attained nanomolar affinity values. As lately outlined by Sluchanko in ref. ^152^, the presence of multivalent sites poses a spiny question related to the stoichiometry of 14-3-3 complexes, which is further amplified in the case of large proteins, such as LRRK2, for which a complete structural analysis in complex with 14-3-3s is still missing. In a recent comparative analysis, Manschwetus et al. investigated the binding affinity shown by each 14-3-3 isoform and confirmed that all but the σ-isoform are able to bind LRRK2, with the highest affinity shown by isoforms -γ and -η^14^. We also showed that 14-3-3γ is able to bind LRRK2. Upon phosphorylation at Ser59 by PAK6, 14-3-3γ monomerizes and dissociates from LRRK2, which, in turn, becomes dephosphorylated at Ser935. Notably, with this mechanism, PAK6 is able to rescue neurite shortening caused by G2019S-LRRK2 in primary neurons^63^.

Ser910/935 are not sites of autophosphorylation, as shown by the fact that kinase-dead variants of LRRK2 only lead to a partial dephosphorylation of these residues and/or LRRK2 re-localization^153^. This implies that alternative kinases, such as PKA^154^, inhibitor of nuclear factor *κB* (I*κ*B)^155^, and casein kinase I alpha (CKIα)^156^ may be involved. Phosphorylation at these sites is compromised in several mutant forms of LRRK2 associated with PD. For example, R1441G and Y1699C, and to a minor extent G2019S, show reduced phosphorylation of Ser935 and concomitant impairment of 14-3-3 binding^154^. Similarly, mutations at R1441 prevent PKA phosphorylation of Ser1444, and with this, the binding of 14-3-3s. Curiously, LRRK2 inhibitors also induce LRRK2 dephosphorylation at Ser910/935 and consequent 14-3-3 release. One possibility is that LRRK2 inhibitors destabilize LRRK2, leading to a reduced amount of the protein and thus explaining their beneficial effects^157^. The pathological reduced binding of LRRK2 to 14-3-3s may lead to different functional outcomes, such as an increased activity of LRRK2^98,158^, re-localization, and accumulation of LRRK2 into cytoplasmic pools reminiscent of those observed in PD patients^147^, or the exclusion of LRRK2 release in exosomes (Fig. 3b, bottom left box)^159^. Indeed, PD rodent models and postmortem PD brains show reduced 14-3-3:LRRK2 interaction and an associated increase in LRRK2 kinase activity^160^. As mentioned, dephosphorylation of Ser910/935 induced by LRRK2 inhibitors is also associated with increased ubiquitination and partial degradation of LRRK2. In accordance, R1441C, Y1699C, and I2020T mutants show dephosphorylation at Ser935, cytoplasmic accumulation, and increased LRRK2 ubiquitination^161^. Also, dissociation of the 14-3-3:LRRK2 complex and re-localization of LRRK2 in the cytoplasmic clusters occurs upon PAK6-dependent phosphorylation of 14-3-3γ (unpublished observations). In accordance, overexpression of 14-3-3θ/τ and direct binding with LRRK2 reduce its kinase activity and reverse neurite shortening observed in G2019S-LRRK2 transgenic mice, while 14-3-3θ/τ inhibition with the competitive peptide inhibitor difopein disrupts its binding to LRRK2, promotes LRRK2 kinase activity, and further increases neurite shortening in G2019S-LRRK2 mice^98^. This suggests that the binding of 14-3-3s to LRRK2 may induce a conformational change, shifting the kinase into its inactive form^158^. Consistent with this, LRRK2 inhibitors reverse the neurite shortening induced by difopein, suggesting that the regulation of kinase activity by 14-3-3s is required for this protective effect on neurite dynamics^98^.

#### Interaction between 14-3-3s and α-Synuclein

Together with isoforms -β and -γ, α-Syn defines the family of synucleins in mammals. α-Syn is a small (14.4 kDa), intrinsically disordered protein mainly localized at the presynaptic terminals and involved in the regulation of synaptic function and plasticity^162^. So far, five rare autosomal dominant mutations (A30P, E46K, H50Q, G51D, and A53T) as well as genomic copy number variations have been identified and associated with the pathogenesis of PD, suggesting that alterations in the gene may be causally related to neuronal degeneration and LB formation^163–169^. α-Syn contains 4 tyrosine residues (Tyr39, Tyr125, Tyr133, and Tyr136) that can be cross-linked by oxidants and nitrating agents and form insoluble aggregates^170^. In animal models, the overexpression of α-Syn or the injection of α-Syn fibril seeds leads to cellular and tissue alterations reminiscent of those observed in PD patients, such as loss of dopaminergic neurons, neurite swelling, and activation of microglia and peripheral inflammatory cells^171–173^. α-Syn and 14-3-3s share regions of homology, and α-Syn interacts with some targets of 14-3-3s, such as BAD and PKC, although with differences in affinity and subcellular localization^174^. α-Syn has one major site of phosphorylation, Ser129, which is partially phosphorylated in basal conditions^175^, and one additional phosphorylation site, Ser87, which seems to be specific to the α-isoform^176^. Both of these sites can be phosphorylated by casein kinases CK1 and CK2 as well as polo-like kinase 2 (PLK2), while Tyr125 can be substrate of Src kinases^176,177^. Phosphorylation at Ser129 is drastically and selectively increased in the brains of patients affected by synucleinopathies and is faithfully mirrored in several animal models^178,179^. Phosphorylation at this residue works as a molecular switch to regulate α-Syn activity and subcellular localization as well as its binding to membranes, ions, and proteins^180^. LBs are enriched in α-Syn phosphorylated at Ser129, in both humans and experimental models^178^. Of note, the phosphorylation of α-Syn is not a prerequisite for 14-3-3 binding. Indeed, low stoichiometric amounts of 14-3-3η can interact with unphosphorylated α-Syn and reduce the formation of toxic α-Syn aggregates in vitro^17^. This is in agreement with studies suggesting that 14-3-3θ/τ may act as a chaperone and protect α-Syn from folding into a pathogenic conformation or even reshape already misfolded α-Syn. In vitro and cellular experiments have shown that 14-3-3 isoforms -ɛ, -γ, and -θ/τ reduce α-Syn inclusions and protect from rotenone-induced toxicity^16,181,182^. 14-3-3θ/τ also reduces α-Syn aggregation and neuronal loss in cellular and in vivo fibril models, while difopein accelerates α-Syn aggregation and toxicity^52^ and^55^.

α-Syn possesses prion-like behavior and can therefore transfer from one cell to another through tunneling nanotubes, exosomes, or other cellular processes^183^. This prion-like behavior occurs also in PD patients, in which healthy neuronal transplants show host-derived misfolded α-Syn a few years after the graft^184,185^. This physical transmission may explain the spreading of α-Syn to different brain regions over time, which remarkably overlaps with the progression of clinical symptoms in PD patients, as postulated by Braak and colleagues^186–188^. 14-3-3θ/τ may impede cell-to-cell transmission of pathologic α-Syn, as demonstrated by studies using paracrine cellular models. Despite promoting total α-Syn release, overexpression of 14-3-3θ/τ reduces the release of misfolded α-Syn species, thus preventing its diffusion and internalization by surrounding neurons (Fig. 3b, top right box). Similarly, manipulation of 14-3-3s also alters the propagation of α-Syn across synapses in the fibril model, as determined by the use of multi-chamber, microfluidic devices^52^.

What leads to 14-3-3 dysfunction in PD is unclear. One possibility is that excess α-Syn may promote 14-3-3 dysregulation, and different hypotheses have been formulated on the possible mechanisms. α-Syn might act at the transcriptional level, as supported by the observation that 14-3-3 isoforms -ɛ, -γ, and -θ/τ are downregulated in cells and mice overexpressing α-Syn^16,127^. Also, by interacting with 14-3-3s, α-Syn could sequester 14-3-3s and reduce its inhibitory action on pro-apoptotic partners, such as BAD, thus leading to neuronal vulnerability^129,189^. Soluble α-Syn complexes may induce specific dopamine toxicity through the generation of reactive oxygen species and the withdrawal of 14-3-3s^189^.

Reduction in functional 14-3-3 levels by either transcriptional dysregulation or sequestration would thereby limit the ability of 14-3-3s to regulate the activity of its partners, including α-Syn and LRRK2. In addition, α-Syn overexpression increases 14-3-3θ/τ phosphorylation at Ser232 in several PD models, and increased Ser232 phosphorylation is observed in insoluble fractions from postmortem human PD brains^128,130^. Ser232 phosphorylation is associated with a loss of 14-3-3θ/τ’s neuroprotective effects against rotenone and MPTP^128^. Aberrant Ser232 phosphorylation could potentially disrupt the interaction of 14-3-3θ/τ with α-Syn and LRRK2, and thereby lead to toxicity due to increased α-Syn misfolding and LRRK2 kinase activity.

#### Interaction between 14-3-3s and Parkin

Parkin is a 52 kDa E3 ubiquitin–protein ligase involved in several cellular pathways, including the degradation of misfolded proteins through the ubiquitin–proteasome system and the regulation of mitochondrial homeostasis^190^. Parkin structure is characterized by a sequential string of domains: a ubiquitin-like domain (Ubl) at its N terminus followed by two zinc-coordinating RING (really interesting new gene)-like domains (RING0, RING1), one IBR (in between RING finger) domain, a linker region, and a final RING2 domain^191^. Several mutations of Parkin have been associated both with autosomal recessive early-onset parkinsonism, usually characterized by the absence of LB formation, and with sporadic forms of PD^192–194^. Parkin interacts with α-Syn and participates in many pathways associated with its regulation, likely contributing to the degradation of the misfolded protein and the rescue of the toxic effects^195,196^. Parkin also associates and co-localizes with 14-3-3η in LBs. This interaction is mediated by the linker region of Parkin, resulting in the inhibition of Parkin self-ubiquitination as well as the ubiquitination of its cellular targets (Fig. 3b, bottom right box). Curiously, the wild-type form of α-Syn (but not the mutant A30P or A53T) can disrupt the binding between 14-3-3η and Parkin by sequestering 14-3-3η^15^. Moreover, Parkin and 14-3-3s are both involved in the removal of damaged mitochondria, a process called mitophagy. Under autophagy-inducing stress conditions, Parkin ubiquitinates proteins in the outer membrane of mitochondria. This begins a cascade of events that culminate with the lack of mammalian target of rapamycin complex 1 (mTORC)-dependent phosphorylation of the transcription factor EB (TFEB). Unphosphorylated TFEB cannot bind to cytoplasmic 14-3-3s and is therefore translocated to the nucleus where it induces the transcription of different lysosomal and autophagic genes^197,198^.

All together, these observations suggest that a better understanding of the regulatory pathways controlling the interactions of 14-3-3s with LRRK2, α-Syn, and Parkin will be crucial to get further insights into the pathogenesis of PD and may serve as a potential target in the search for new therapeutic strategies.

### Stabilizers and inhibitors of 14-3-3s: translational opportunities?

Given the large amount of client proteins and the consequent variety of cellular activities involved, it is reasonable to consider 14-3-3s as prospective targets for future drug therapies.

The first step in the development of new drugs relies on the identification of candidate targets. In this context, computational algorithms based on position-specific scoring matrix, support vector machine, or artificial neural networks, such as Scansite 4.0, EML, NetPhorest, ANIA, and 14-3-3 Pred, are now available to the community and have become of use to predict sequence-specific binding motifs^199–204^. Once identified, these targets and their interaction with the protein of interest need to be validated. NMR and crystallographic data have provided major insights about the spatial conformation of 14-3-3 isoforms and their target-bound structures^31^. Although technical limitations have restricted the analyses mainly to 14-3-3s associated with short peptide fragments, these have been critical in determining the physical-chemical principles ruling 14-3-3 PPIs. Even more, it is on the basis of these crystallographic analyses that the first small molecules have been identified and successively optimized^205^. More recently, the advent of computational algorithms has assigned new boundaries to our prediction potential. As mentioned, Stevers and coworkers developed a thermodynamic model to estimate the binding affinities of peptides derived from the multiple 14-3-3-binding sequences of CFTR and LRRK2. In particular, with this model they were able to predict how the mutation/deletion of specific phospho-sites (e.g., pSer910 or pSer935 in LRRK2) could have affected the binding of the mentioned proteins to 14-3-3s^42^. Models like this combined with classical crystal structural analyses will accelerate the identification and targeting of key residues whose modification might increase the efficacy and specificity of therapeutic small molecules. While specificity is always a sought-after requirement in drug development, this is particularly true—and difficult to achieve—in the case of hot-spot proteins, such as 14-3-3s. Indeed, because of the high structural conservation of the binding groove and the occurrence of similar binding motifs in hundreds of target proteins, the risk of incurring unwanted side effects is potentially high. As mentioned, however, the specificity of the target binding does not depend on the conserved amphipathic groove, but it rather relies on the surrounding client-specific residues. Based on this observation, secondary binding sites can be pinpointed and specifically targeted to modulate 14-3-3s interactome^206^. While in some cases (e.g., 14-3-3:LRRK2 or 14-3-3:α-Syn) a stabilization of the 14-3-3:target binding is desirable, in others a de-stabilization would be preferred, locking the choice to the specific context. In both cases, PPI modulators may work either as competitors—contending the same binding spot with all the proteins that possess a region with similar structural features- or as allosteric modulators—changing the affinity of a protein for its binding partner in a more indirect way. Each of these solutions comes with pros and cons. Indeed, while competitive inhibitors are geared toward rational design strategies, they lack specificity, since they might block the interaction of the protein of interest (e.g., 14-3-3) with any of its partners. On the opposite, PPI stabilizers may be more difficult to frame, but they do ensure more specificity, since their glue-like action relies on the exclusive interaction platform offered by two interacting partners^207–209^.

So far, some promising results have been obtained by small molecules acting either as competitive inhibitors (e.g., R18, difopein) or as non-competitive stabilizers (fusicoccanes) of 14-3-3:target binding. R18 has been initially isolated from a phage display library; it can enter in the groove and affect the binding of the associated target^210^. As an example, R18 can inhibit the binding of 14-3-3s with Raf-1 or FOXO3a, ultimately leading to cell apoptosis and suggesting a possible strategy in the treatment of cancer^210,211^. As mentioned, R18 interferes in the interaction between 14-3-3s and PKA, leading to the dissociation of its regulatory and catalytic subunits^95^. Similarly to R18, difopein (composed by two repeats of the R18 peptide separated by a flexible linker) may induce apoptosis^212,213^. Interestingly, difopein has been used to inhibit the binding between 14-3-3s and LRRK2, proving that this interaction is necessary to LRRK2 activity^98^ and to prevent LRRK2 ubiquitination^161^. However, these peptides are non-specific, since they bind to the groove of all the isoforms and regardless of the associated target. Nevertheless, these first approaches proved that 14-3-3s may represent a druggable target and paved the way to the discovery of an increasing number of 14-3-3 PPI inhibitors with better specificity (for an extensive review, see ref. ^208^).

Fusicoccanes are a family of natural small molecules that recognize the binding groove and stabilize 14-3-3:client complexes. However, their accessibility is constrained by specific residues in the binding motif, thus restricting their activities to a limited range of 14-3-3 targets^214^. FC-A and the closely related Cotylenin A (Cot-A) have been used to promote the interactions between 14-3-3s and some specific targets. FC-A can stabilize the interaction between 14-3-3β and CFTR by selectively interacting with a secondary motif of CFTR. This strengthens the binding to 14-3-3β and promotes the transportation of CFTR^151^. FC-A also promotes neurite outgrowth in spinal cord and optic nerve injury models by stabilizing the interaction of 14-3-3s with the stress response regulator GCN-1^96^. Cot-A has been shown to work as an anti-tumor agent when used in combination with other molecules such as Vincristine or anti-EGFR antibody^215^. Cot-A selectively binds to the pSer233 and pSer259 residues of Raf, which are also putative 14-3-3 binding sites. The Raf:14-3-3ζ interaction at these Ser residues (but not to pSer621) results in Raf inhibition. The concomitant coupling of Cot-A further stabilizes this interaction and prevents Raf pro-survival activity^216^.

Interestingly, the presence of a C12-dehydroxy allows Cot-A to work as a stabilizer of target proteins binding either motif I or II. In contrast, hydroxylation occurring on FC-A prevents such interaction and classifies FC-A as a motif III-specific stabilizer^208^. These structural observations boosted the search for chemical modifications aimed at the development of semi-synthetic molecules endowed with functional specificity. Among these, FC-THF (modified with a tetrahydrofuran ring), ISIR-005 (which lacks C12 hydroxylation), and FC aglycones (where the glycosyl group has been replaced by a hydrogen) have been tested for their ability to stabilize 14-3-3:protein complexes^217–219^. High-throughput screenings of synthetic peptides have identified Epibestatin and Pyrrolidone-1 as compounds able to specifically stabilize the interaction between 14-3-3s and the proton pump PMA2, although their binding modality differs from each other^220^. Structural analysis identifying FC-A derivatives that increase neurite outgrowth revealed that these semi-synthetic FC-derived molecules have dual functionality to promote certain protein client interactions while disrupting others through structural shifts in the 14-3-3 binding pocket^221^.

All together these observations show that 14-3-3s could be legitimate candidates for drug discovery. In particular, the use of crystal structures and bioinformatics tools together with new technologies such as dynamic combinatorial chemistry, covalent tethering, and structure-activity relationship associated with NMR will greatly help the identification of new small molecules with enhanced therapeutic potential^222–225^.

## Conclusions

Despite the lack of a signature catalytic activity, 14-3-3s are involved in a myriad of pathways critical to the maintenance of cellular homeostasis. By binding to specific phospho-motifs (although with some exception), 14-3-3s interact with a variety of intracellular proteins, including enzymes, transcription factors, and cytoskeletal proteins^2^. For this reason, 14-3-3s have acquired increasing interest over the years, boosted by evidence that altered 14-3-3 function is observed in numerous pathologies, such as cancer and neurodegenerative diseases, including PD. As a matter of fact, 14-3-3s interact with different proteins involved in the pathogenesis of PD, including LRRK2, α-Syn, and Parkin. Manipulation of 14-3-3:target protein interactions to interfere with some of the pathways involved in PD pathogenesis may serve as a new therapeutic approach. However, the search for drugs aimed at interfering with 14-3-3:target interaction must take into account the need for specificity. In this respect, the great advances made in the past decades in the field of X-ray crystallography, NMR spectrometry, and electron microscopy, as well as the implementation of databases and prediction algorithms, will be critical to exploit the full therapeutic potential of 14-3-3s.
